# “GeSn Rule-23”—The Performance Limit of GeSn Infrared Photodiodes

**DOI:** 10.3390/s23177386

**Published:** 2023-08-24

**Authors:** Guo-En Chang, Shui-Qing Yu, Greg Sun

**Affiliations:** 1 Department of Mechanical Engineering, Advanced Institute of Manufacturing with High-Tech Innovations, National Chung Cheng University, Chia-Yi 62102, Taiwan; 2 Department of Electrical Engineering, University of Arkansas, Fayetteville, AR 72701, USA; syu@uark.edu; 3 Department of Engineering, University of Massachusetts-Boston, Boston, MA 02125, USA; greg.sun@umb.edu

**Keywords:** dark current, GeSn alloys, infrared, responsivity, silicon photonics, CMOS, sustainability

## Abstract

Group-IV GeSn photodetectors (PDs) compatible with standard complementary metal–oxide-semiconductor (CMOS) processing have emerged as a new and non-toxic infrared detection technology to enable a wide range of infrared applications. The performance of GeSn PDs is highly dependent on the Sn composition and operation temperature. Here, we develop theoretical models to establish a simple rule of thumb, namely “GeSn−rule 23”, to describe GeSn PDs’ dark current density in terms of operation temperature, cutoff wavelength, and Sn composition. In addition, analysis of GeSn PDs’ performance shows that the responsivity, detectivity, and bandwidth are highly dependent on operation temperature. This rule provides a simple and convenient indicator for device developers to estimate the device performance at various conditions for practical applications.

## 1. Introduction

The Group-IV GeSn material system is under extensive development for low-cost and high-performance infrared (IR) photodetectors (PDs) for a wide spectrum of applications covering military, communication, and thermal vision [1,2]. While the present market-dominated IR PDs made of compound semiconductors such as InGaAs, InSb, HgCdTe, PbSe, PbS, etc. offer good quantum efficiencies, they are less compatible with the standard complementary metal–oxide-semiconductor (CMOS) processing, leading to high cost and complex fabrication. By contrast, the CMOS compatibility of GeSn PDs makes them ideal for monolithic integration with electronics on the same Si or silicon-on-insulator (SOI) chip for seamlessly manufacturing in modern CMOS foundries, allowing for the development of low-cost, high-performance, complex, and functional image systems [3,4]. In addition, GeSn alloys offer a wide range of bandgap tunability by adjusting the Sn composition [5,6], thereby permitting expansion of their direct-gap absorption edge from ~1500 nm to short-wave IR (SWIR) range (1.5–3 μm), mid-wave infrared (MWIR) range (3–8 μm), and even long-wave infrared (LWIR) (8–14 μm). Most importantly, the presence of the indirect conduction band enables a unique momentum–space carrier separation scheme to enable high-performance photodetection [7,8]. These unique advantages have encouraged the development of various types of GeSn PDs [8,9,10,11,12,13,14,15,16,17,18,19,20,21,22,23,24,25] with remarkable photodetection range up to 4600 nm [25]. More recently, a comprehensive theoretical study of GeSn PDs has indicated that the performance of GeSn PDs when reaching material maturity is comparable with, and even better than, market-dominated IR PDs [6], showing great promise for low-cost and high-performance IR detection.

IR PDs usually require cryogenic cooling to suppress dark current in order to reach high performance for practical photodetection. In addition, the device performance such as dark current, cutoff wavelength, responsivity, and detectivity are strongly dependent on the operation temperature as well as alloy composition. Thus, from the viewpoint of device and system developers, a rule of thumb is extremely useful to describe the performance of IR PDs in terms of operation temperature and alloy composition to achieve desired performance for meeting the requirement of various applications. Such simple relationships have been established to evaluate the performance of IR PDs including Mercury–Cadmium–Telluride (MCT) PDs (rule-07) [26] and extended-wavelength InGaAs PDs (IGA-rule-17) [27]. However, such a simple rule of thumb has not been developed for GeSn PDs so far. Here, for the first time, a heuristic rule is proposed and established for GeSn PDs with various Sn compositions and operation temperatures as GeSn-rule 23, where the prefix GeSn stands for GeSn PDs and the postfix 23 represents the year 2023, to evaluate the performance of GeSn PDs for the device and system developers to advance the GeSn IR PD technology.

The rest of the paper Is organized as follows: the structure of the GeSn *p-i-n* PD under investigation is described in Section 2; the theoretical models of temperature-dependent bandgap energies for analyzing the cutoff wavelength in terms of Sn composition and operation temperature are presented in Section 3; the theoretical models for evaluating dark current density and the establishment of GeSn-rule-23 are presented in Section 4; the theoretical models of temperature-dependent absorption coefficient and optical responsivity are shown in Section 5; the detectivity and noise-equivalent power in terms of Sn content and operation temperature are discussed in Section 6; the analysis of temperature-dependent bandwidth of GeSn PDs is given in Section 7; and finally the conclusion is summarized in Section 8.

## 2. GeSn Device Structure

**Figure 1:**
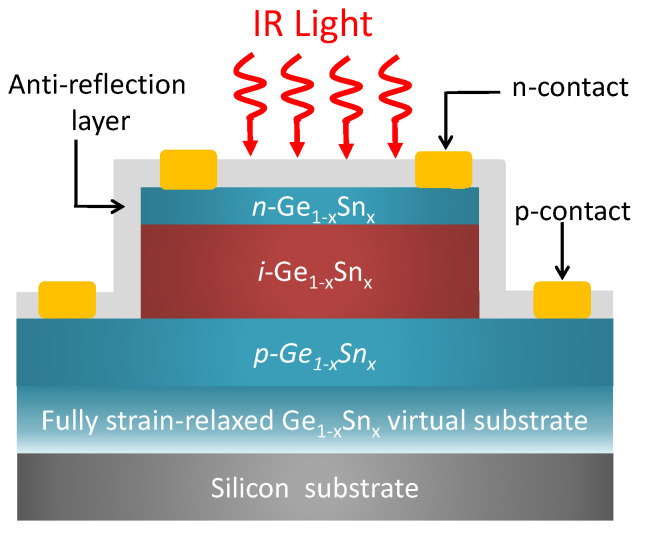
Schematic diagram of normal-incidence GeSn *p*−*i*−*n* homojunction photodetector on a Si (001) substrate with a GeSn virtual substrate.

To establish the GeSn PDs’ fundamental performance, here we shall consider a general normal-incidence Ge_1−x_Sn_x_ *p*−*i*−*n* homojunction PDs with a circular mesa, as shown in Figure 1, so the optical response of the Ge_1−x_Sn_x_ PD is independent of the incident light’s polarization. The Ge_1−x_Sn_x_ *p-i-n* diode is grown on a (001) silicon substrate via a fully strain-relaxed Ge_1−x_Sn_x_ virtual substrate (VS), so the entire Ge_1−x_Sn_x_ *p*−*i*−*n* stack is strain-free. It is noted that the inhomogeneity of Sn content in the material may significantly affect the material properties [28]. In this study, we assume the Sn content in the Ge_1−x_Sn_x_ *p-i-n* stack is uniform. The intrinsic Ge_1−x_Sn_x_ layer can convert the incident IR photons to electron-hole pairs via optical absorption, which are then swept across the *p*−*i*−*n* junction and collected as electrical currents. To achieve high responsivity and suppress tunneling dark current, a thick intrinsic Ge_1−x_Sn_x_ layer is necessary. Here, we set the thickness of the intrinsic Ge_1−x_Sn_x_ layer to be *t*_i_ = 3000 nm to enhance optical absorption and quantum efficiency. The thickness of the *n*-Ge_1−x_Sn_x_ is kept thin to be *t*_n_ = 100 nm to enhance the optical absorption by the intrinsic Ge_1−x_Sn_x_ layer. On the other hand, the thickness of the *p*- Ge_1−x_Sn_x_ is fixed to *t*_p_ = 500 nm to ensure sufficient etching tolerance for mesa definition. The doping concentrations in the *n*−Ge_1−x_Sn_x_ and *p*−Ge_1−x_Sn_x_ are set to $N_{a}=1\times 10^{19}{\mathrm{cm}}^{-3}$ and $N_{d}=1\times 10^{19}{\mathrm{cm}}^{-3}$, respectively, because high doping concentrations can help suppress diffusion dark currents and thus enhance detectivity [5]. (For different thicknesses and doping concentrations of the layers, the device performance can also be evaluated using our theoretical models). We make several assumptions in order to evaluate their achievable performance. First, we shall assume that the entire Ge_1−x_Sn_x_ *p-i-n* stack is defect-free as the defects can be properly confined in the Ge_1−x_Sn_x_ VS [8]. The diameter of the GeSn diode mesa is set to *D* = 50 µm, which is significantly larger than the wavelength of interest, so the diffraction effect is negligible. On top of the GeSn is an antireflection layer which can minimize the reflection loss and thereby enhance quantum efficiency. The parameters for GeSn alloys used in this study were obtained from linear interpolation between these of Ge and α-Sn [29], and their dependences on wavelength (electrical frequency) are neglected.

## 3. Temperature-Dependent Bandgap Energies and Cutoff Wavelength

The cutoff wavelength (*λ*_c_) of the Ge_1−x_Sn_x_ PDs is determined by optical absorption edges of the *i*−Ge_1−x_Sn_x_ active layer. For Ge_1−x_Sn_x_ alloys, the optical absorption has two contributors, the direct-gap and indirect-gap interband absorption, owing to the proximity of the Γ- and L-valley conduction band (CB). However, the indirect−gap interband absorption is much weaker than the direct-gap one [6] because of the need of additional phonons for momentum conservation. As a result, the cutoff wavelength of the Ge_1−x_Sn_x_ PDs is dominated by the direct-bandgap ($E_{g}^{\Gamma }$) via the expression $\lambda_{c}=1.24/E_{g}^{\Gamma }$. The temperature− and composition−dependent direct bandgap energy of Ge_1−x_Sn_x_ alloys can be calculated using the Varshni equation [30,31,32]

(1) $$E_{g}^{\Gamma }(x,T)=E_{g}^{\Gamma }(x,T=0)-\frac{\alpha (x)\times T^{2}}{T+\beta (x)}$$

(2) $$\begin{array}{l} E_{g}^{\Gamma }(x,T=0)=E_{g}^{\Gamma }(\mathrm{Ge},T=0)\times (1-x)+E_{g}^{\Gamma }(\mathrm{Sn},T=0)\times x-b_{\Gamma }x(1-x) \end{array}$$

where *α* and *β* are the Varshni parameters for Ge_1−x_Sn_x_ alloys, $E_{g}^{\Gamma }(T=0)$ is the Γ-valley bandgap energy at *T* = 0 K, and $b_{\Gamma }=2.46\;\mathrm{eV}$ is the bowing parameter [30]. The Varshni parameters for Ge_1−x_Sn_x_ alloys are obtained by linear interpolation from these of Ge and α-Sn given in Table 1.

**Figure 2:**
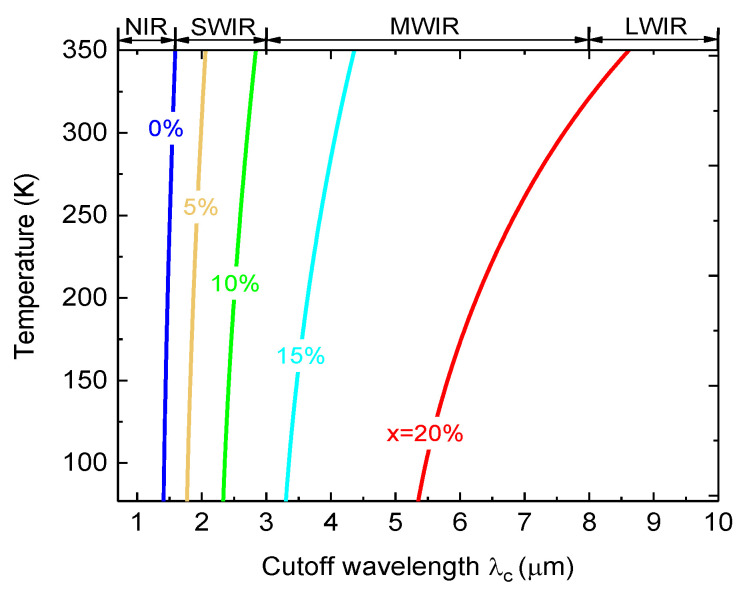
Calculated cutoff wavelength versus operation temperature for GeSn PDs with different Sn contents.

The relationships between the operation temperature and cutoff wavelength for GeSn PDs with different Sn compositions are shown in Figure 2. For a fixed Sn content, the cutoff wavelength increases with increasing temperature. For pure Ge (x = 0%), the cutoff wavelength can only reach NIR. As the Sn content increases, the cutoff wavelength of Ge_1−x_Sn_x_ PDs significantly redshifts attributed to the bandgap shrinkage owing to the incorporation of Sn, and can reach SWIR, MWIR, and even LWIR range with a sufficiently high Sn content. These results show that Ge_1−x_Sn_x_ PDs can operate in different IR spectral ranges by adjusting the Sn content for different applications.

## 4. Dark Current Density and GeSn-Rule 23

For defect−free Ge_1−x_Sn_x_ PDs, the dark current density is dominated by minority carrier diffusion currents [6]. The diffusion dark current density at zero bias under short-base approximation can be calculated using [6]

(3) $$J_{s}(x,T)=q [\frac{D_{p}(x,T)}{t_{p}}p_{n0}(x,T)+\frac{D_{n}^{\Gamma }(x,T)}{t_{n}^{}}n_{p0}^{\Gamma }(x,T)+\frac{D_{n}^{L}(x,T)}{t_{n}^{}}n_{p0}^{L}(x,T)]$$

where *q* is the elementary charge; $D_{p}$, $D_{n}^{L}$, and $D_{n}^{L}$ are the diffusion coefficients for holes, and electrons in the Γ−CB and L−CB, respectively; $p_{n0}$ is the minority hole density in the *n*−GeSn region; $n_{p0}^{\Gamma }$ and $n_{p0}^{L}$ are the minority electron densities in the Γ− and L−CB in the *p*−GeSn region. The diffusion coefficient can be converted from mobility (*µ*) via the Einstein relationship *D = µkT/q* with *k* being the Boltzmann constant [32]. The minority carrier concentrations in the doped layers can be linked to the intrinsic carrier concentrations and doping concentrations via $(n_{p0}^{\Gamma }+n_{p0}^{\Gamma })=n_{i}^{2}/N_{a}$ and $p_{n0}=n_{i}^{2}/N_{d}$. The intrinsic carrier concentration (*n*_i_) in GeSn alloys can be calculated using [6,32]

(4) $$n_{i}^{2}={\left(n_{i}^{\Gamma }+n_{i}^{L}\right)}^{2}=N_{C}^{\Gamma }N_{V}\exp\left(-\frac{E_{g}^{\Gamma }}{kT}\right)+N_{C}^{L}N_{V}\exp\left(-\frac{E_{g}^{L}}{kT}\right)$$

(5) NCΓ=22πmΓ*kTℏ232

(6) NCL=22πmL*kTℏ232

where ℏ is the reduced Planck constant, $m_{\Gamma }^{*}$ and $m_{L}^{*}$ are the electron effective masses in the Γ− and L−CB, respectively, $m_{h}^{*}$ is the hole effective mass in the valence band, which are taken from a 30−band full−zone k·p model [33]. The electron mobility in the Γ−CB ($\mu_{e,\Gamma }^{0}$) and L−CB ($\mu_{e,L}^{0}$) and the hole mobility ($\mu_{h}^{0}$) (in units of cm^2^V^−1^s^−1^) for intrinsic Ge_1−x_Sn_x_ at *T* = 300 K can be expressed as [34,35]

(7) $$\mu_{e,\Gamma }^{0}(x,T=300K)=306,230-483,516x$$

(8) $$\mu_{e,L}^{0}(x,T=300K)=3800+4456x$$

(9) $$\mu_{h}^{0}(x,T=300K)=1800+33,750x$$

The temperature-dependent mobility can be approximated with the power law $\mu \propto T^{-p}$ [36], where *p* is a constant. Owing to the lack of experimental data for GeSn alloys, the coefficients are approximated by these of Ge (*p* = 1.66 for electrons and *p* = 2.33 for holes [36]). With the mobilities, the minority mobility can be estimated using [37,38]

(10) $$\mu_{e}=\frac{\mu_{e}^{0}}{1+\sqrt{N_{a}\times 10^{-17}}}$$

(11) $$\mu_{h}=\frac{\mu_{h}^{0}}{1+\sqrt{N_{d}\times 2.1\times 10^{-17}}}$$

where *N*_a_ and *N*_d_ are the doping concentrations (in units of cm^−3^) in the *p*− and *n*−GeSn regions, respectively. With the dark current density, the *R_0_A* product can be obtained using [32]

(12) $$R_{0}^{}A=\frac{kT}{q}\times \frac{1}{J_{s}}$$

**Figure 3:**
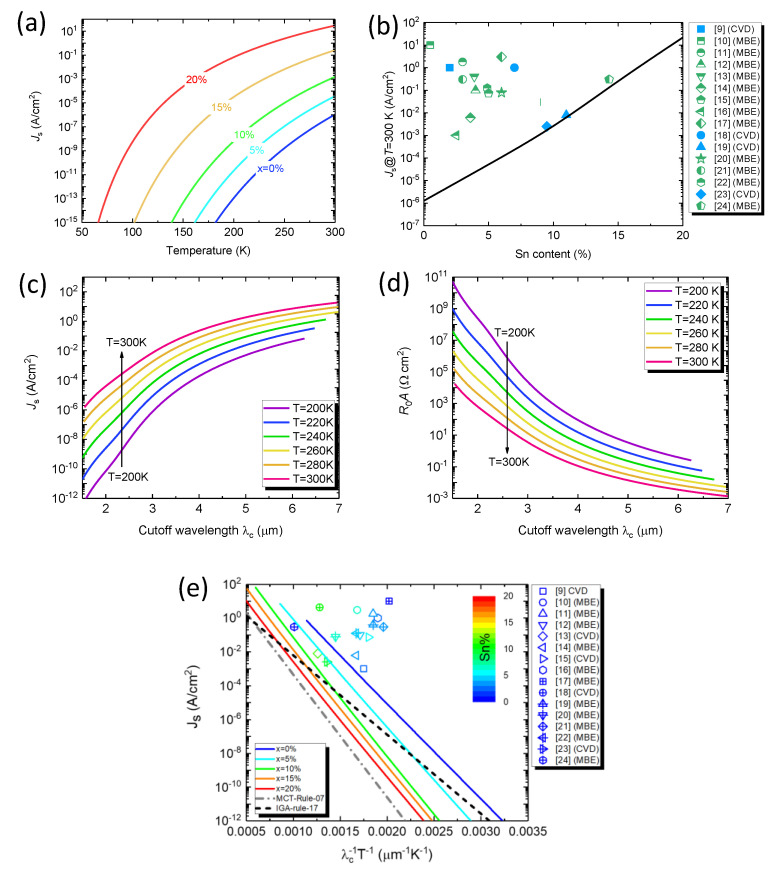
Calculated GeSn−rule−23 performance for GeSn PDs. (**a**) Calculated dark current density as a function of temperature. (**b**) Calculated dark current density at *T* = 300 K for GeSn PDs as a function of Sn content. Experimental dark current densities taken from Refs. [9,10,11,12,13,14,15,16,17,18,19,20,21,22,23,24] are also shown for comparison. Calculated (**c**) dark current density, and (**d**) *R*_0_*A* product of the GeSn PDs as a function of cutoff wavelength in the temperature range of *T* = 200–300 K. (**e**) Calculated GeSn−rule−23 performance by means of dark current density as a function reciprocal of the product cutoff wavelength and temperature (solid lines) compared with reported experimental data (scatters) in the literature [9,10,11,12,13,14,15,16,17,18,19,20,21,22,23,24]. MCT rule−07 (dashed−dotted line) [26] and IGA−rule−17 (dashed line) [27] and are also depicted for comparison.

Figure 3a shows the calculated dark current density as a function of operation temperature for Ge_1−x_Sn_x_ PDs with different Sn compositions. For a fixed Sn content, the dark current increases with increasing temperature owing to higher intrinsic carrier concentration. In addition, the dark current density also increases with increasing Sn content owing to higher intrinsic carrier concentration as a result of reduced bandgap energy. Figure 3b shows the calculated dark current density compared with selected experimental data from the reported Ge_1−x_Sn_x_ PDs operated at −1 V bias voltage and *T* = 300 K [9,10,11,12,13,14,15,16,17,18,19,20,21,22,23,24]. It can be found that the calculated dark current density is generally 2–3 orders of magnitude smaller than experimental data at *T* = 300 K. The results suggest that it is possible to significantly improve the dark current density by continuously improving the material quality. Figure 3c,d shows the calculated dark current density and *R*_0_*A* product as a function of cutoff wavelength at various temperatures in the range of *T* = 200–300 K, respectively. At a fixed temperature, Ge_1−x_Sn_x_ PDs require a higher Sn composition to extend the cutoff wavelength, causing larger dark current density and smaller *R*_0_*A*. At higher temperatures, the dark current density goes up while *R_0_A* product goes down. It is noted that the use of three-stage thermoelectric (TE) coolers can decrease the operation temperature of IR PDs to 210 K [39]. Thus, it is anticipated that decreasing the operation temperature using TE coolers can effectively suppress the dark current density of Ge_1−x_Sn_x_ PDs. Figure 3e depicts the calculated dark current density as a function of product reciprocal of the product cutoff wavelength and temperature product (*λ_c_T*)^−1^ for Ge_1−x_Sn_x_ PDs with various Sn compositions. Also plotted in Figure 3e are the selected experimental data from the reported Ge_1−x_Sn_x_ PDs operated at −1 V bias voltage [9,10,11,12,13,14,15,16,17,18,19,20,21,22,23,24] as well as the MCT rule-07 [26] and IGA-rule-17 [27]. It can be seen that, for a fixed Sn composition, the dark current density decreases with increasing (*λ_c_T*)^−1^ because lower temperatures suppress the dark current density. The experimental data observed in Ge_1−x_Sn_x_ PDs show dark current densities about 1–3 orders of magnitude higher than the calculated performance. This discrepancy is likely attributed to (1) the residual compressive strain in the Ge_1−x_Sn_x_ active layer that enlarges the bandgap energy and thus blueshifts the cutoff wavelength, and (2) the defects in the Ge_1−x_Sn_x_ active layer that induce defect−related dark currents. These results suggest that there is considerable room to reduce the dark current density of Ge_1−x_Sn_x_ PDs by improving the material quality. In comparison with IGA−rule−07, it is found that Ge_1−x_Sn_x_ PDs with low Sn compositions (<5%) have higher dark current density than the E−InGaAs PDs for small (*λ_c_T*)^−1^. As the Sn composition increases, the dark current density decreases, and eventually becomes comparable to, or even lower than, the E-InGaAs PDs, suggesting superior performance can be obtained with Ge_1−x_Sn_x_ PDs operating in longer-wavelength and higher-temperature conditions. Relative to MCT−rule−07, however, the Ge_1−x_Sn_x_ PDs exhibit higher dark current densities than MCT PDs.

We now establish GeSn-rule-23 based on our calculation results. The relationship between the dark current density, cutoff wavelength, and operation temperature can be described by the empirical expression [26,27]

(13) $$J_{s}(x,T)=J_{0}\times \exp\left(-C^{\prime }\frac{1.24q}{k}\times \frac{1}{\lambda_{c}T}\right)$$

where *C′* and *J*_0_ are the fitting parameters, which can be obtained using the calculated results in Figure 3e and the results are listed in Table 2. These results offer device and system developers to easily evaluate the performance of GeSn PDs at different operation conditions. The dark current density of Ge_1−x_Sn_x_ PDs at *T* = 300 K (*J*_s_@*T* = 300 K) is also listed in Table 2 for comparison. *J*_s_@*T* = 300 K significantly increases with increasing Sn contents as a result of narrower bandgap energy.

## 5. Temperature-Dependent Optical Absorption and Responsivity

Next, we calculate the temperature-dependent optical absorption coefficient and responsivity of the GeSn PDs. For GeSn alloys, both direct−gap and indirect−gap interband transitions contribute to optical absorption. However, the indirect−gap interband transition is much weaker than the indirect-gap because additional phonons are necessary to conserve momentum. Thus, the absorption coefficient is dominated by the direct-gap absorption coefficient. The direct-gap optical absorption coefficient by the direct-gap transitions can be calculated using the Fermi’s golden rule taking into account the Lorentzian lineshape function and the nonparabolicity effect as [4]

(14) $$\alpha \left(E\right)=\frac{\pi \hbar q^{2}}{n_{r}c\varepsilon_{0}m_{0}^{2}E}\sum_{m}\int \frac{2dk}{{\left(2\pi \right)}^{3}}{\left|\hat{q}\cdot p_{CV}\right|}^{2}\times \frac{\gamma /2\pi }{{ [E_{C\Gamma }\left(k\right)-E_{m}\left(k\right)-E]}^{2}+{\left(\gamma /2\right)}^{2}}$$

where *n*_r_ is the refractive index of GeSn alloys; *c* is the velocity of light in vacuum; *ɛ*_0_ is the free space permittivity; *m*_0_ is the rest mass of electron; *ω* is the angular frequency of incident light; *E* is the incident photon energy; ${\left|\hat{q}.p_{CV}\right|}^{2}=m_{0}E_{P}/6$ is the momentum matrix with *E*_p_ denoting the optical energy parameter; *γ* is the full-width-at-half-maximum (FWHM) of the Lorentzian lineshape function. $E_{C\Gamma }(k)$ and $E_{m}(k)$ the electron energy in the Γ-CB and hole energy in the valence band, respectively, which are calculated using a multi-band k·p model presented in Refs. [4,40].

**Figure 4:**
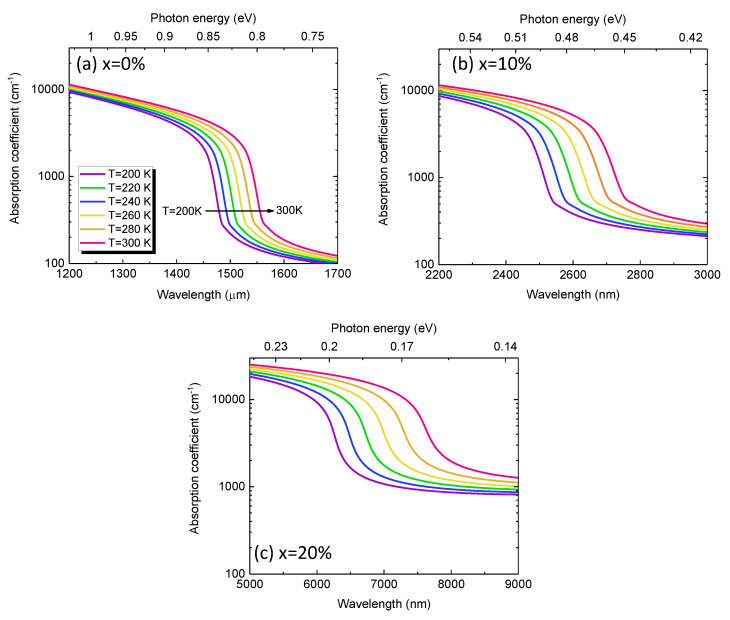
Calculated absorption coefficient spectra of GeSn alloys with Sn contents of (**a**) *x* = 0%, (**b**) *x* = 10%, and (**c**) *x* = 20% in the temperature range of *T* = 200–300 K.

Figure 4 depicts the calculated absorption coefficient spectra of GeSn alloys with different Sn contents in the temperature range of *T* = 200–300 K. For a fixed Sn content and temperature, the absorption coefficient gradually decreases with increasing wavelength, followed by a sharp decrease near the direct-bandgap energy. It can also be observed that the absorption spectra redshift as the temperature increases because of the reduced direct bandgap energy. Note that the direct-gap absorption coefficient is related to the joint density-of-state (JDOS), which is proportional to $\sqrt{E-E_{g}}$ [31]. As a result, the absorption coefficient at a fixed wavelength increases with increasing temperature. As the Sn content increases, the absorption coefficient significantly redshifts owing to the narrowed bandgap energy caused by Sn alloying. As a result, the absorption coefficient in the MWIR region can be significantly enhanced, thereby enabling efficient MWIR photodetection.

With the absorption coefficient, we can then calculate the optical responsivity ($R_{\lambda }$) of the GeSn PDs using [31]

(15) $$R_{\lambda }=\frac{q\lambda }{hc}\eta_{i}\left(1-R_{\mathrm{refl}}\right) [1-\exp\left(-\alpha t_{i}\right)]$$

where *η*_i_ is the internal quantum efficiency and its value is assumed to be *η*_i_ = 100%; and *R*_refl_ is the reflectivity of the top surface of the GeSn PDs and its value is assumed to be zero as the anti–reflection coating can minimize the reflection.

**Figure 5:**
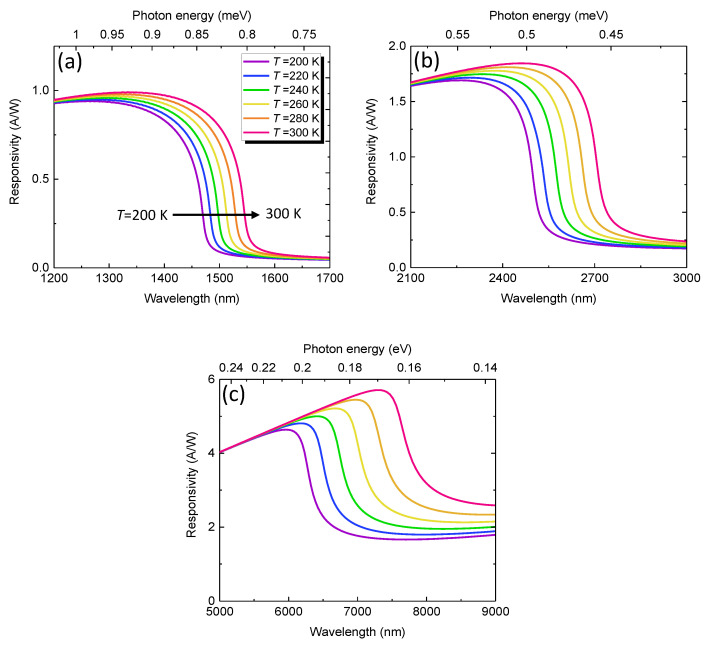
Calculated responsivity spectra of GeSn PDs with Sn contents of (**a**) *x* = 0%, (**b**) *x* = 10%, and (**c**) *x* = 20% in the temperature range of *T* = 200–300 K.

Figure 5 depicts the calculated optical responsivity spectra of the GeSn PDs with various Sn contents in the temperature range of *T* = 200–300 K. For pure Ge PDs (*x* = 0%), as shown in Figure 5a, the responsivity increases with increasing wavelength, and then sharply decreases near the direct–gap absorption edge, correspondingly to the cutoff wavelength of the PD. With an increase in the operation temperature, the cutoff wavelength exhibits a redshift, indicating a wider photodetection range. As the Sn content increases to 10%, as shown in Figure 5b, the cutoff wavelength is significantly extended to ~2600 nm. Meanwhile, the optical responsivity significantly increases because the reduced photon energy per photon leads to more photons for a watt of light energy. As the Sn content further increases to 20%, the cutoff wavelength is further extended to ~7000 nm with enhanced optical responsivity, enabling sensitive MWIR photodetection.

## 6. Temperature-Dependent Detectivity and Noise-Equivalent Power

The figure-of–merit for the performance of PDs includes not only dark current density, but also detectivity. Next, we calculate detectivity of Ge_1−x_Sn_x_ PDs in terms of Sn content and temperatures. With *R*_0_*A* and responsivity *R*_λ_, we can obtain the specific detectivity ($D_{\lambda }^{*}$), one of the most important figure-of-merits used to characterize PDs’ performance, as [6,31]

(16) $$D_{\lambda }^{*}(\lambda )=\frac{R_{\lambda }(\lambda )}{2\sqrt{qJ_{0}}}$$

The corresponding metric noise-equivalent power (NEP), which represents the minimum detectable power per square root bandwidth, can be computed as [31]

(17) $$NEP=\frac{R_{\lambda }{\left(A\times \Delta f\right)}^{1/2}}{D_{\lambda }^{*}(\lambda )}$$

where *A*(=$\pi D^{2}/4$) is the area of the photosensitive region of the GeSn PDs and Δf is the bandwidth of the PD.

**Figure 6:**
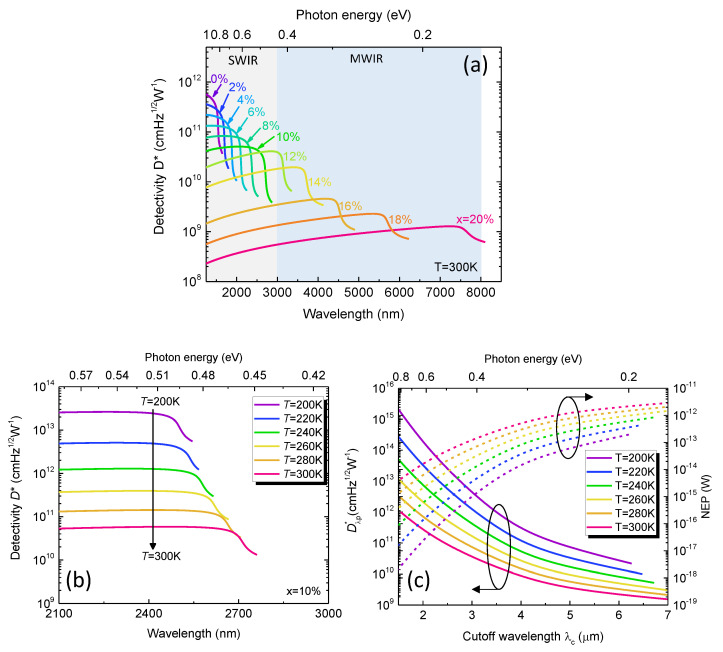
(**a**) Calculated specific detectivity spectra of GeSn PDs with various Sn contents at *T* = 300 K. (**b**) Calculated specific detectivity spectra for Ge_0.9_Sn_0.1_ PD in the temperature range of *T* = 200–300 K. (**c**) Calculated peak detectivity and NEP as a function of cutoff wavelength in the temperature range of *T* = 200–300 K.

Figure 6a shows the calculated specific detectivity spectra of the GeSn PDs with various Sn content at *T* = 300 K. For a fixed Sn content, $D_{\lambda }^{*}$ increases with increasing wavelength, followed by a rapid decrease near the direct-gap absorption edge. As a result, the peak detectivity ($D_{\lambda_{p}}^{*}$) is obtained at a wavelength of about $\lambda_{p}=0.9\lambda_{c}$. For *x* = 0, a high $D_{\lambda }^{*}\sim 3\times 10^{11}{\mathrm{cmHz}}^{1/2}W^{-1}$ is achieved at *λ*~1500 nm. As the wavelength increase,$D_{\lambda }^{*}$ drops rapidly and becomes negligible near the direct–gap absorption edge because incident IR photons cannot be fully absorbed by the GeSn active region. As the Sn content increases, the detectivity spectrum is extended to longer wavelengths owing to the reduced direct bandgap, and can fully cover the entire SWIR (MWIR) spectral range with a Sn content of ~12% (~20%). However, $D_{\lambda }^{*}$ also decreases as a result of increased dark current density. Figure 6b shows the calculated specific detectivity spectra for GeSn PDs with *x* = 10% in the temperature range of *T* = 200–300 K. As the temperature decreases, $D_{\lambda }^{*}$ significantly increases owing to the suppressed dark current density. Meanwhile, the photodetection range exhibits a blueshift as a result of increased direct bandgap energy. (For other Sn contents, similar results are obtained.) Figure 6c shows the peak detectivity $D_{\lambda }^{*}$ (which is defined as the detectivity at the wavelength $\lambda_{p}=0.9\lambda_{c}$) and the corresponding NEP normalized to a bandwidth of Δf=1 Hz as a function of cutoff wavelength in the temperature range of *T* = 200–300 K. At a fixed temperature, $D_{\lambda_{p}}^{*}$ decreases and NEP increases with increasing cutoff wavelength of the Ge_1−x_Sn_x_ PDs owing to higher Sn compositions. Meanwhile, $D_{\lambda_{p}}^{*}$ decreases and NEP increases at higher operation temperatures owing to increased dark current density.

## 7. Temperature-Dependent Bandwidth

We then investigated the temperature-dependent 3-dB bandwidth of the GeSn PDs. The 3–dB bandwidth of PDs is usually governed by transit–time delay and RC time delay. The transit–time delay limited bandwidth (*f*_T_) can be calculated using [41,42]

(18) $$f_{T}=0.45\frac{v_{S}}{t_{i}}$$

where *v*_s_ is the carrier saturation velocity. The carrier saturation velocity at *T* = 300 K can be calculated using [42,43]

(19) $$v_{S}=\sqrt{\frac{\Delta E}{m^{*}\sigma_{s}N_{s}L_{me}}}$$

where $\Delta E=hc_{s}/a_{\mathrm{GeSn}}$, *c*_s_ is the velocity of sound in the GeSn material which can be obtained by $c_{s}=\sqrt{G/\rho }$ with *G* being the shear modulus and *ρ* being the density, *a*_GeSn_ is the bulk lattice constant of GeSn alloys, m* is the conductivity effective mass. The temperature-dependent saturation velocity can be approximated with [44]

(20) $$v_{S}(T)=\frac{v_{S}(T=300\;K)}{\left(1-\sigma )+\sigma (T/300\right)}$$

where σ is a constant. Owing to the lack of experimental data for GeSn alloys, the coefficient for GeSn alloys is approximated by that of Ge (σ = 0.45 for electrons and 0.39 for holes).

On the other hand, the RC-time delay limited bandwidth can be calculated using [41,42]

(21) $$f_{RC}=\frac{1}{2\pi RC}$$

where *C* is the capacitance of the GeSn junction, which can be calculated using $C={\varepsilon A/t}_{i}$; and *R* is the load resistance which is set to the standardized RF impedance of *R* = 50 Ω. Note that the permittivity of the materials is a function of temperature. Owing to the lack of experiment data for GeSn alloys, the permittivity is obtained by linear interpolation of these Ge and α-Sn. The temperature–dependent permittivity of Ge is taken from Ref. [45]. For α-Sn, the temperature–dependent permittivity is not available, so we approximate it by the value at *T* = 300 K ($\varepsilon_{r}=24$). As the Sn content in this study is not very high, we believe that this is still a good approximation. With the transit–time-delay and RC-delay bandwidths, the total 3-dB bandwidth of the PD can be obtained using [41,42]

(22) $$f_{3\mathrm{dB}}=\sqrt{\frac{1}{f_{T}^{-2}+f_{\mathrm{RC}}^{-2}}}$$

With the 3-dB bandwidth, the response time (*t*_r_) can be obtained using [42,46]

(23) $$t_{r}=\frac{0.35}{f_{3\mathrm{dB}}}$$

**Figure 7:**
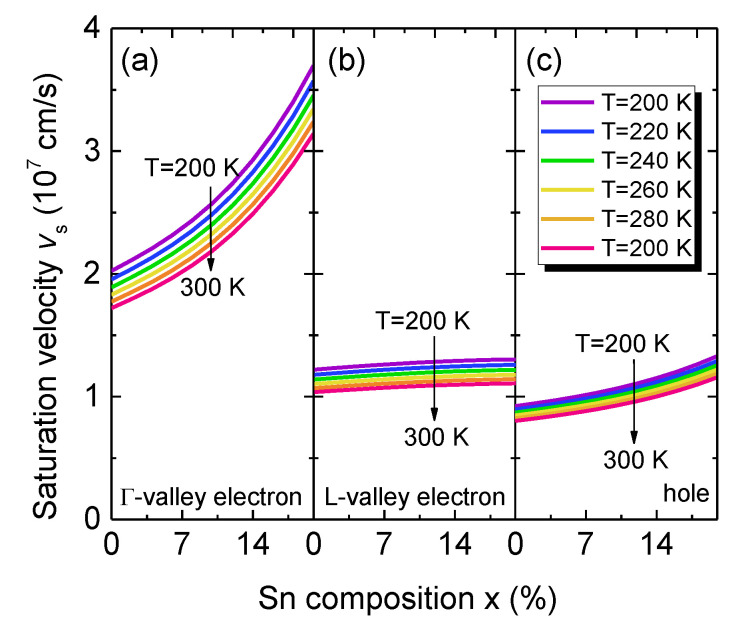
Calculated (**a**) Γ–CB, (**b**) L-CB electron and (**c**) hole saturation velocities as a function of Sn content in the temperature range of *T* = 200–300 K.

Figure 7 shows the calculated saturation velocities of electrons in the Γ–CB and L–CB and holes in the valence band as a function of Sn content at various temperatures. For a fixed temperature, the saturation velocities increase with increasing Sn content, because of the increased carrier mobilities. As the temperature increases, the electron and hole saturation velocities decrease because of the reduced mobilities. For electron saturation velocities, electrons in the Γ–CB have much higher saturation velocities then these in the L–CB, because of their much smaller effective mass (m* = 0.045 − 0.166*x* + 0.043*x*^2^ for Γ–CB electrons and for m* = 0.566 − 0.449*x* + 1.401*x*^2^ for L-CB electrons [33]). The hole saturation velocity is much smaller than the electron ones, because of the much larger effective masses. Thus, when electron–hole pairs are created by the absorption of incident photons in the GeSn active region, holes require longer time to transit through the active region than electrons. As a result, the transit–time–delay–limited bandwidth is dominated by the transition time of holes.

**Figure 8:**
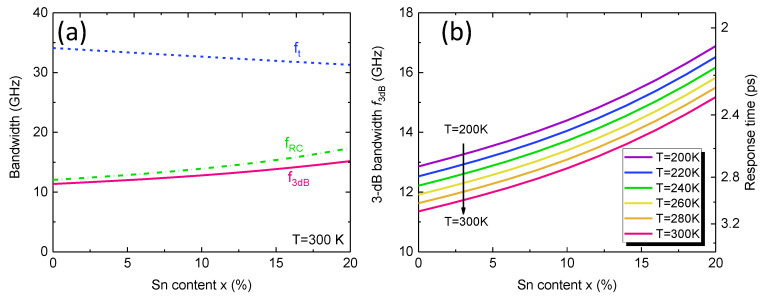
(**a**) Calculated transit-time-delay-limited bandwidth, RC-delay-limited bandwidth, and 3-dB bandwidth of GeSn PDs as a function of Sn content at *T* = 300 K. (**b**) Calculated 3-dB bandwidth and response time as a function of Sn content at various temperatures.

Figure 8a depicts the calculated transit–time–delay–limited, RC–delay–limited, and 3–dB bandwidth of the GeSn PD as a function of Sn content at *T* = 300 K. The transit–time–delay–limited bandwidth decreases with increasing Sn content as a result of increased carrier saturation velocity. On the other hand, the RC–delay–limited bandwidth decreases with increasing Sn content owing to the increased permittivity of the GeSn active layer. However, transit–time–delay-limited bandwidth is significantly lower than the RC–delay–limited bandwidth owing to the thick GeSn active layer. As a result, the 3–dB bandwidth is dominated by transit–time–delay–limited bandwidth. As the Sn content increases, the 3–dB bandwidth increases slightly and can be greater than 10 GHz, corresponding to a response time of <3.5 ps, indicating the capacity of high–speed operation. Figure 8b shows the calculated 3–dB bandwidth and response time of the GeSn PDs as a function of Sn content in the temperature range of *T* = 200–300 K. With an increase in temperature, the 3–dB bandwidth slightly decreases, but can remain >10 GHz.

## 8. Conclusions

We have developed GeSn-rule-23 for the purpose of evaluating the performance limit of Ge_1−x_Sn_x_ IR PDs. This rule establishes the relationship between the cutoff wavelength and operating temperature for GeSn PDs with different Sn compositions. Fitting parameters that describe the dependence of dark current density on cutoff wavelength and temperature are determined. In comparison with the experimental data obtained so far from the reported Ge_1−x_Sn_x_ PDs, this study suggests that their performance has significant room to improve. In addition, temperature-dependence analysis of the optical responses indicates that the optical responsivity increases, while the detectivity and 3–dB bandwidth decrease at higher temperatures. The GeSn-rule-23 is expected to provide useful guidelines for device and system developers to select proper Sn compositions and operation temperature to achieve the desired device performance to meet the requirement of practical applications.

## Figures and Tables

**Table 1:** Varshni parameters for GeSn alloys [30].

Symbol	Ge	Sn
$E_{g}^{\Gamma }(T=0)$ (eV)	0.89	−0.39
α (eV K^−1^)	$5.8\times 10^{-4}$	$-7.94\times 10^{-7}$
*β* (K)	296	11

**Table 2:** Fitted parameters from the GeSn-rule-23 and these for and MCT-rule-07 [26] IGA-rule-17 [27].

Symbol	C′	*J*_0_ (A/cm^2^)	*J*_0_@*T* = 300 K (A/cm^2^)
GeSn (*x* = 0%)	0.907	$2.00\times 10^{6}$	$1.27\times 10^{-6}$
GeSn (*x* = 5%)	1.005	$1.66\times 10^{6}$	$5.09\times 10^{-5}$
GeSn (*x* = 10%)	1.129	$1.01\times 10^{6}$	$2.19\times 10^{-3}$
GeSn (*x* = 15%)	1.202	$3.65\times 10^{5}$	0.269
GeSn (*x* = 20%)	1.386	$1.13\times 10^{5}$	21.92
IGA-rule-17 [26]	0.757	277.0	$7.548\times 10^{-5}$
MCT-rule-07 [27]	1.163	8367.0	--

## Data Availability

The data presented in this study are available upon request from the corresponding author. The data are not publicly available due to commercial privacy policy.
